# Antarctic Krill Oil Diet Protects against Lipopolysaccharide-Induced Oxidative Stress, Neuroinflammation and Cognitive Impairment

**DOI:** 10.3390/ijms18122554

**Published:** 2017-11-28

**Authors:** Ji Yeon Choi, Jun Sung Jang, Dong Ju Son, Hyung-Sik Im, Ji Yeong Kim, Joung Eun Park, Won Rak Choi, Sang-Bae Han, Jin Tae Hong

**Affiliations:** College of Pharmacy and Medical Research Center, Chungbuk National University, 194-31 Osongsaemgmyeong 1-ro, Osong-eup, Heungdeok-gu, Cheongju 28160, Chungbuk, Korea; cjy8316@hanmail.net (J.Y.C.); chvictory07@hanmail.net (J.S.J.); sondj1@hotmail.com (D.J.S.); ihs311@naver.com (H.-S.I.); ronevans@naver.com (J.Y.K.); wlwl83@naver.com (J.E.P.); cwonr@sk.com (W.R.C.); shan@chungbuk.ac.kr (S.-B.H.)

**Keywords:** neuroinflammation, amyloidogenesis, oxidation, nuclear factor-κB, krill oil

## Abstract

Oxidative stress and neuroinflammation are implicated in the development and pathogenesis of Alzheimer’s disease (AD). Here, we investigated the anti-inflammatory and antioxidative effects of krill oil. Oil from *Euphausia superba* (Antarctic krill), an Antarctic marine species, is rich in eicosapentaenoic acid (EPA) and docosahexaenoic acid (DHA). We examined whether krill oil diet (80 mg/kg/day for one month) prevents amyloidogenesis and cognitive impairment induced by intraperitoneal lipopolysaccharide (LPS) (250 µg/kg, seven times daily) injections in AD mice model and found that krill oil treatment inhibited the LPS-induced memory loss. We also found that krill oil treatment inhibited the LPS-induced expression of inducible nitric oxide synthase (iNOS) and cyclooxygenase-2 (COX-2) and decreased reactive oxygen species (ROS) and malondialdehyde levels. Krill oil also suppresses IκB degradation as well as p50 and p65 translocation into the nuclei of LPS-injected mice brain cells. In association with the inhibitory effect on neuroinflammation and oxidative stress, krill oil suppressed amyloid beta (1–42) peptide generation by the down-regulating APP and BACE1 expression in vivo. We found that eicosapentaenoic acid (EPA) and docosahexaenoic acid (DHA) (50 and 100 µM) dose-dependently decreased LPS-induced nitric oxide and ROS generation, and COX-2 and iNOS expression as well as nuclear factor-κB activity in cultured microglial BV-2 cells. These results suggest that krill oil ameliorated impairment via anti-inflammatory, antioxidative, and anti-amyloidogenic mechanisms.

## 1. Introduction

Dietary intervention with marine products, including marine-derived oils, has been widely used during the past decades. Furthermore, within the previous three decades, natural marine products have shown many promising activities against inflammation, cancer, infectious diseases and neurological disorders [1]. The consumption of marine products prevents neurodegenerative processes and maintains cognitive capacities in the elderly [2]. Of these products, krill oil can regulate lipid metabolism, inflammation, and oxidative stress [3]. The shrimp-like *Euphausia superba* (Antarctic krill) is one of the most important Antarctic marine species [4]. Previous studies have demonstrated that krill oil has anti-inflammatory and antioxidative effects due to its eicosapentaenoic acid (EPA) and docosahexaenoic acid (DHA) contents, which can be absorbed very quickly and cross the blood–brain barrier (BBB) [5]. It was also reported that EPA and DHA, which are found in animal-based sources of omega-3 fats, play a significant role in lowering tumor necrosis alpha (TNF-α), interleukin 1 beta (IL-1β), and prostaglandin E_2_ levels [6]. Additionally, krill oil is rich in vitamin A and E, and the carotenoid astaxanthin, which is likely stable and resistant to oxidation [7]. Therefore, high levels of these components make krill oil more superior than fish oil in terms of its biological effects [8].

Oxidative stress and inflammation are the two major processes in the development of Alzheimer’s disease (AD). Oxidative stress is a condition in which oxidant generation overwhelms antioxidant defenses and is largely implicated in the pathogenesis of many neurologic and psychiatric, diseases including AD [2]. Increased oxidative stress leads to damage to lipids, DNA, and proteins, and thus causes a functional decline in neurons [9]. Oxidative stress has been proposed to upregulate amyloid beta (Aβ) peptide generation via induction of β- and γ-secretase activity [10]. Hydrogen peroxide (H_2_O_2_) in human neuroblastoma cells reportedly enhances BACE1 expression and Aβ accumulation, eventually causing significant cell damage [11,12]. Additionally, AD brain exhibit oxidative stress-mediated injury since Aβ peptides increase superoxide anion production in the brain [13]. Thus, synaptic loss and increased number of extracellular Aβ peptides could be associated with oxidative brain damage [14]. Brain inflammation is also a pathological hallmark of the AD. The activated microglial cells produce inflammatory mediators and accumulate around amyloid plaques in the brains of individuals with the AD, and have been implicated in promoting neurodegeneration [15]. Chronically activated glia can kill adjacent neurons by releasing highly toxic products such as reactive oxygen species (ROS), nitric oxide (NO), and complement factors, thereby enhancing APP production and amyloidogenic processing [16]. Exposure of lipopolysaccharide (LPS) has cognitive-behavioral consequences due to Aβ aggregation in the hippocampus and pro-inflammatory reactions in response to oxidative damages [17]. Therefore, the study of protective compounds that inhibit oxidative pathways and inflammatory responses is an aspect of further research for treating neurodegenerative diseases.

Nuclear factor-kappa B (NF-κB) is a redox transcription factor that influences the levels of oxidative stress in neurons [18,19]. Expression of several inflammatory genes such as inducible nitric oxide synthase (iNOS) and cyclooxygenase-2 (COX-2), as well as inflammatory cytokines, can be regulated by NF-κB activation [20]. It is also known that oxidative stress can activate NF-κB in several disease statuses. Moreover, the promoter of neuronal BACE1, a limiting enzyme producing Aβ, has NF-κB DNA consensus sequences [21]. Epidemiologic studies have demonstrated that the anti-inflammatory and antioxidative therapies could decrease the risk of the AD by reducing NF-κB activity [22]. Thus, blocking NF-κB can facilitate AD management by reducing neuroinflammation, oxidative stress, and amyloidogenesis [23].

In the present study, we investigate whether Antarctic krill oil has antioxidative and anti-inflammatory properties as well as anti-amyloidogenic property against LPS-induced memory dysfunction in cultured neuronal macrophages and in vivo mice models.

## 2. Results

### 2.1. Krill Oil Treatment Attenuates LPS-Induced Cognitive Impairment

**Figure 1:**
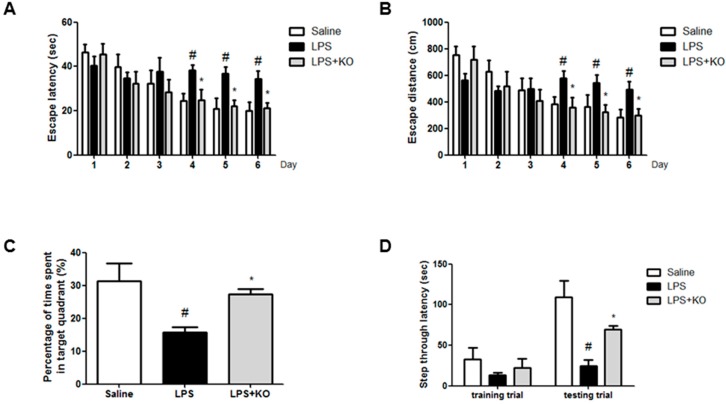
Effect of Krill oil on memory impairment. To investigate the effect of Krill oil in LPS-induced memory impairment, we performed: a water maze test (**A**,**B**); a probe test (**C**); and a step-through type passive avoidance test (**D**). Memory function was determined by the escape latencies (**A**, s) and distance (**B**, cm) for 5 days, and time spent in the target quadrant (**C**, %) in the probe test after administration of LPS. Each value is mean ± Standard deviation (SD) from eight mice. Group differences were analyzed by one-way ANOVA followed by Bonferroni’s post-hoc analysis. ^#^ Significantly different from control group (*p* < 0.05). * Significantly different from LPS-treated group (*p* < 0.05).

Effect of krill oil on cognitive and memory improvement was estimated using the water maze and passive avoidance tests. We investigated the ability of mice to learn locations and perform spatial memory recall through escape latency and measuring the distance in the water maze. The LPS-injected mice learned more slowly than control mice and krill oil-treated mice. Krill oil-treated mice exhibited a reduction in escape latency over the training period (Figure 1A). Krill oil-treated mice also showed a shorter escape distance (Figure 1B) compared with LPS-injected mice. After the final day of the water maze test, we performed a probe test to calculate the time spent in the target quadrant zone, thereby testing for the maintenance of memory. Krill oil-treated mice spent much more time in the quadrant zone than LPS-injected mice (Figure 1C). Then, through the passive avoidance test, we tested the mice to assess for how long they can remember the locations. Although there was no significant difference in the learning pattern, krill oil-treated mice recorded increased step-through latency compared with the LPS-injected mice group (Figure 1D).

### 2.2. Krill Oil Treatment Inhibits the Accumulation of Aβ Peptides, Amyloidogenesis, and NF-κB Activation

**Figure 2:**
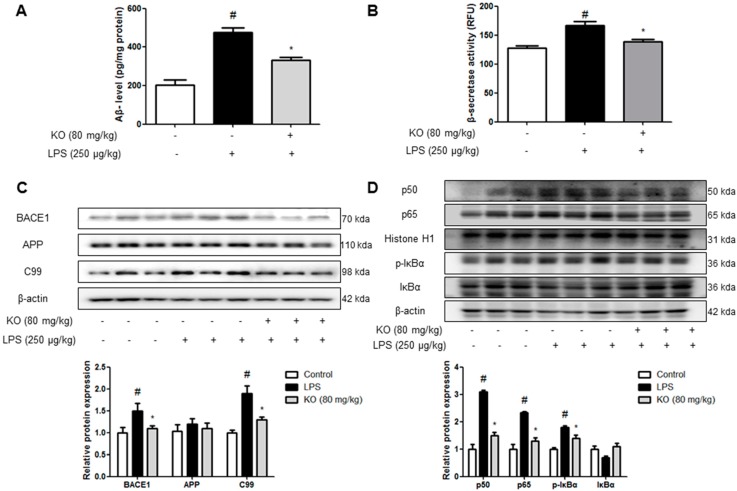
Effect of Krill oil on the LPS-induced amyloidogenesis and NF-κB activity in mice brain. The levels of Aβ_1–42_ in mice brain (*n* = 5) were measured by ELISA (**A**). The activity of β-secretase in mice brain (*n* = 5) was investigated using assay kit (**B**). The expression of APP, BACE1 and C99 were detected by Western blotting using specific antibodies in the mouse brain (**C**). Phosphorylation of IκB, and p50 and p65 translocation were detected by Western blotting using specific antibodies in mice brain; β-actin and Histone H1 protein were used as an internal control (**D**). For the cropped images, samples were run in the same gels under same experimental conditions and processed in parallel. The graphs under Western blotting are the relative protein expression of three bands. Each band is representative of three experiments. Group differences were analyzed by one-way ANOVA followed by Bonferroni’s post-hoc analysis. ^#^ Significantly different from control group (*p* < 0.05). * Significantly different from LPS-treated group (*p* < 0.05).

To investigate whether Aβ deposition by immunohistochemical analysis was paralleled with Aβ level in the brain, quantitative analyses of Aβ level was performed using ELISA. Aβ level in the brains of LPS-injected mice was significantly higher than that of control mice, but it was reduced in the krill oil-treated mice brains (Figure 2A). Because Aβs are produced by activated β-secretase, we measured the activity of β-secretase in the hippocampus. The activity of β-secretase was increased in the brains of LPS-injected mice, while the activity was significantly decreased in krill oil-treated mice brains (Figure 2B). To confirm whether krill oil influenced amyloidogenesis inhibition in the brain, we performed the Western blot assay. LPS-elevated expression of APP, BACE1, and C99 was significantly decreased by krill oil treatment (Figure 2C). NF-κB activity is implicated in amyloidogenesis and neuroinflammation. Thus, due to p50, p65, and IκB phosphorylation, NF-κB activation was confirmed. Phosphorylation of IκB and translocation of p50 and p65 were significantly decreased by the treatment of krill oil (Figure 2D). 

### 2.3. Krill Oil Treatment Inhibits Neuroinflammation

**Figure 3:**
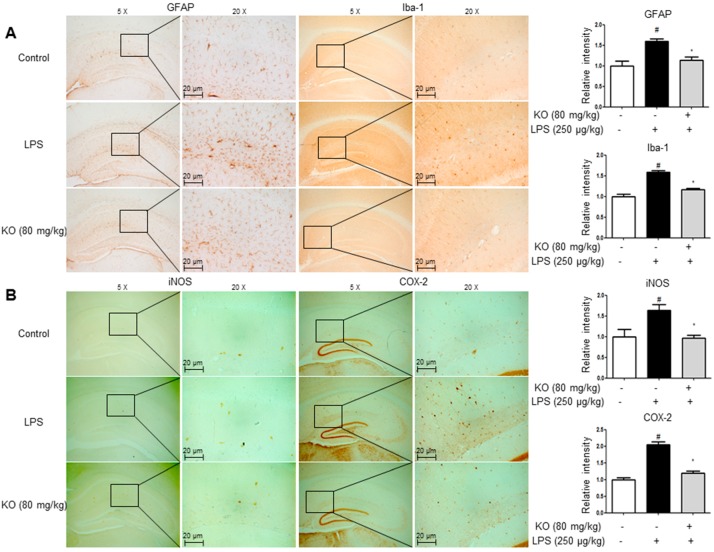
Effect of Krill oil on LPS-induced neuroinflammation in the mouse brain. Immunostaining of GFAP, Iba-1, iNOS, and COX-2 proteins in the hippocampus were performed in 20 µm-thick sections of mice brain with specific primary antibodies and the biotinylated secondary antibodies (**A**,**B**). Similar patterns were observed in five mice brain. Group differences were analyzed by one-way ANOVA followed by Bonferroni’s post-hoc analysis. ^#^ Significantly different from control group (*p* < 0.05). * Significantly different from LPS-treated group (*p* < 0.05).

**Figure 4:**
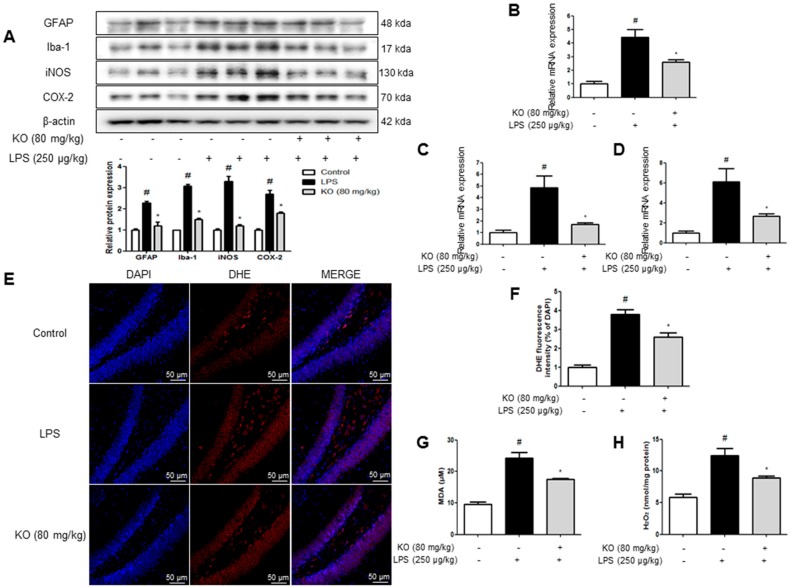
Effect of Krill oil on the LPS-induced neuroinflammation and oxidative stress in the mouse brain. The expression of GFAP, Iba-1, iNOS, and COX-2, were detected by Western blotting using specific antibodies in the mice brain. Each blot is representative of three experiments (**A**). For the cropped images, samples were run in the same gels under same experimental conditions and processed in parallel. The graphs under Western blotting are the relative protein expression of three bands. Each band is representative of three experiments. mRNA levels of IL-6 (**B**); IL-1β (**C**); and TNF-α (**D**) were detected by qRT-PCR in brain tissues (*n* = 5). Intracellular superoxide radical production was measured by dihydroethidium in the brain. The brain sections were double stained with DHE (red) and DAPI staining (blue) (**E**). Similar patterns were observed in five mice brain. The graph is a quantification of the DHA fluorescent signal in the brain tissues (**F**). MDA (**G**); and hydrogen peroxide level (**H**) were assessed by using a specific detection kit as described in Methods (*n* = 5). Values measured from each group of mice were calibrated by the amount of protein and expressed as mean ± SD. Group differences were analyzed by one-way ANOVA followed by Bonferroni’s post-hoc analysis. ^#^ Significantly different from control group (*p* < 0.05). * Significantly different from LPS-treated group (*p* < 0.05).

We performed immunohistochemistry and Western blotting to detect the expression of GFAP (an astrocyte activation marker), Iba-1 (a microglial cell activation marker), and inflammatory proteins (iNOS and COX-2) in the brain, which consequently indicates the activation of astrocytes and microglia as well as the occurrence of neuroinflammation. The GFAP-reactive cell number and Iba-1-reactive cell number were reduced in krill oil-treated mice as opposed to LPS-injected mice, which showed the much higher number of cells reactive for these marker proteins compared with control mice (Figure 3A). The expression of iNOS and COX-2 was also significantly decreased in the brains of krill oil-treated mice than LPS-injected mice brains (Figure 3B). We also investigated the inhibitory effect of krill oil on neuroinflammation through Western blotting; treatment with LPS elevated the expression of inflammatory proteins (iNOS and COX-2), GFAP, and Iba-1, but the expression was significantly reduced by krill oil treatment (Figure 4A). We investigated expression levels of the pro-inflammatory cytokines-related factors IL-6, IL-1β and TNF-α in brain tissues. Our results suggested that krill oil treatment decreased LPS-induced mRNA levels of IL-6 (Figure 4B), IL-1β (Figure 4C) and TNF-α (Figure 4D) in brain tissues. 

### 2.4. Krill Oil Inhibits LPS-Induced Oxidative Stress

Krill oil decreased superoxide anion production in the mice brain. Intracellular superoxide radical production was measured by dihydroethidium in the brain. Furthermore, another study has shown that local LPS administration contributes to the activation of astroglial or microglial cells in place of this toxin administration. Additionally, it was reported that brain damage can be caused by inflammation and oxidative stress after prolonged exposure to LPS for ≥7 days. The accumulation of excessive intracellular ROS with increased enzymatic sources characterizes oxidative stress. Although the intensity of oxidative stress varies because oxidation usually occurs only for a short time, systemic LPS administration for a prolonged period will damage the brain due to exposure to oxidative stress. The brain sections were double stained with DHE (red) and DAPI (blue) staining. The krill oil-treated mice showed a significant decrease in DHE signal intensity compared with the LPS-injected mice (Figure 4E,F). We also evaluated malondialdehyde (MDA) and H_2_O_2_ levels, which are indicators of oxidative stress. MDA was significantly increased in the brains of LPS-injected mice compared with control mice. However, in contrast to LPS-injected mice, krill oil-treated mice showed lower MDA (Figure 4G) and H_2_O_2_ levels (Figure 4H).

### 2.5. EPA and DHA Prevent LPS-Stimulated Nuclear Translocation of the NF-κB Complex

**Figure 5:**
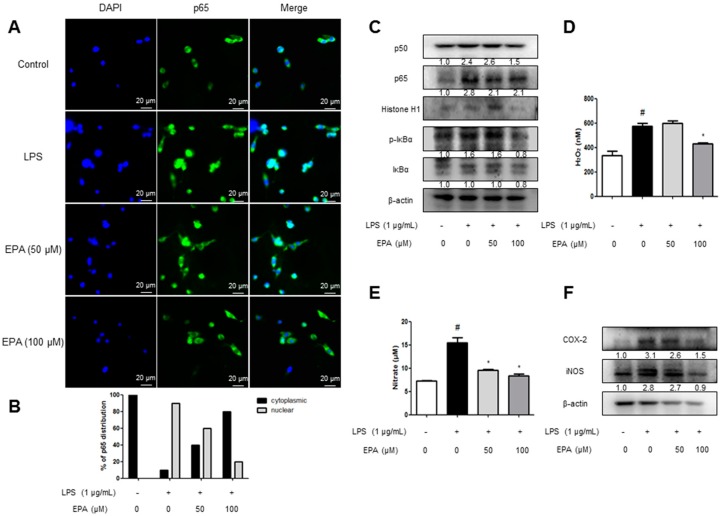
Inhibitory effect of Krill oil on neuroinflammatory responses in microglia cells. To find the effect of anti-oxidative stress, microglial BV-2 cells were treated with 1 µg/mL of LPS and 50, 100 µM of EPA. The cultured microglial BV-2 cells were incubated with anti-p65 (green) and DAPI staining (blue) (**A**). Quantification of p65 subcellular distribution in one representative of three independent experiments (**B**). Fluorescence was developed using Alexa 488-conjugated anti-mouse secondary antibodies. Phosphorylation of IκB, and p50 and p65 translocation were detected by Western blotting using specific antibodies in microglial BV-2 cells. β-actin and Histone H1 protein were used as an internal control (**C**). Hydrogen peroxide level was assessed using a specific detection kit, as described in Methods (**D**) (*n* = 5). NO level was measured in EPA treated microglial BV-2 cells (**E**) (*n* = 5). COX-2 and iNOS proteins were detected by Western blotting using specific antibodies in EPA treated microglial BV-2 cells (**F**). The graphs under Western blotting are the relative protein expression of bands. Each band is representative of three experiments. Group differences were analyzed by one-way ANOVA followed by Bonferroni’s post-hoc analysis. ^#^ Significantly different from control group (*p* < 0.05). * Significantly different from LPS-treated group (*p* < 0.05).

**Figure 6:**
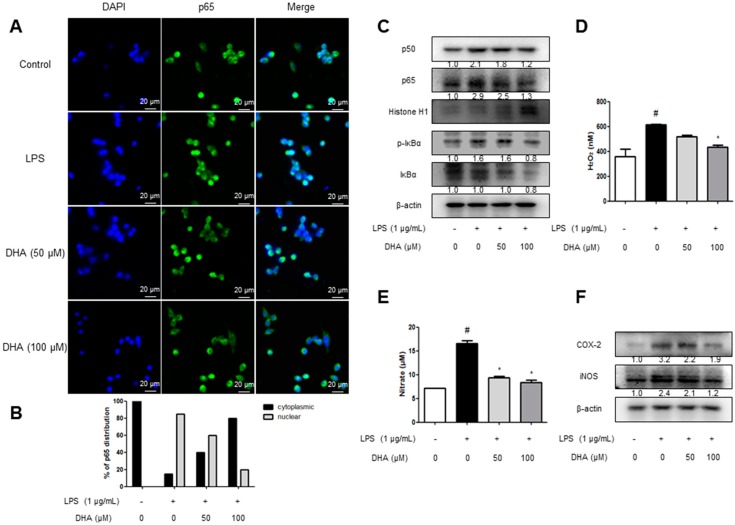
Inhibitory effect of Krill oil on neuroinflammatory responses in microglia cells. To find the effect of anti-oxidative stress, microglial BV-2 cells were treated with 1 µg/mL of LPS and 50, 100 µM of DHA. The cultured microglial BV-2 cells were incubated with anti-p65 (green) and DAPI staining (blue) (**A**). Quantification of p65 subcellular distribution in one representative of three independent experiments (**B**). Fluorescence was developed using Alexa 488-conjugated anti-mouse secondary antibodies. Phosphorylation of IκB, and p50 and p65 translocation were detected by Western blotting using specific antibodies in microglial BV-2 cells. β-actin and Histone H1 protein were used as an internal control (**C**). Hydrogen peroxide level was assessed by using a specific detection kit as described in Methods (**D**) (*n* = 5). NO level was measured in DHA treated microglial BV-2 cells (**E**) (*n* = 5). COX-2 and iNOS proteins were detected by Western blotting using specific antibodies in DHA treated microglial BV-2 cells (**F**). The graphs under Western blotting are the relative protein expression of bands. Each band is representative of three experiments. Group differences were analyzed by one-way ANOVA followed by Bonferroni’s post-hoc analysis. ^#^ Significantly different from control group (*p* < 0.05). * Significantly different from LPS-treated group (*p* < 0.05).

We investigated the effects of EPA and DHA, the components of krill oil, on NF-κB nuclear translocation in microglial BV-2 cells using immunofluorescence imaging. LPS induced nuclear translocation of p65, the NF-κB protein, in 30 min. In contrast, EPA and DHA pretreatment prevented the dose-dependent nuclear translocation of p65 (Figure 5A and Figure 6A). To clarify whether EPA and DHA influenced the inhibition of p65 translocation, we performed Western blotting. We determined NF-κB activation through the detection of p50, p65, and IκB phosphorylation. Phosphorylation of IκB and translocation of p50 and p65 were significantly decreased by the treatment of EPA and DHA (Figure 5B and Figure 6B).

### 2.6. EPA and DHA Prevent LPS-Stimulated Oxidative Stress and Neuroinflammation

We evaluated the H_2_O_2_ level as an indicator of oxidative stress. To elucidate the effect of antioxidative stress, microglial BV-2 cells were treated with 1 µg/mL LPS and 50 and 100 µM of EPA or DHA. The microglial BV-2 cells treated with EPA/DHA showed lower H_2_O_2_ levels (Figure 5C and Figure 6C). Furthermore, it was detected that the NO level was decreased dose-dependently in microglial BV-2 cells (Figure 5D and Figure 6D). Then, we detected the expression of inflammatory proteins (iNOS and COX-2) by Western blotting. The levels of iNOS and COX-2 protein were increased in LPS-treated cells, whereas the expressions were dose-dependently reduced by EPA and DHA treatment (Figure 5E and Figure 6E).

## 3. Discussion

The data from the present study revealed that krill oil supplementation in diet could suppress neuroinflammation, oxidative stress, and amyloidogenesis in LPS-induced AD model. Oxidative stress and neuroinflammatory cascades can lead to neurodegenerative diseases, including AD; thus, the administration of anti-inflammatory and anti-oxidative agents reduces the risk of or delays the neuropathologic features of AD [24,25]. There are different mechanisms related with AD progression. Recently, a series of studies proved that systemic administration of LPS contributes to increased neuroinflammation and oxidative stress along with direct damage of the BBB, thereby causing amyloidogenesis and memory deficiency [26,27]. Furthermore, LPS-induced brain inflammation is accompanied by neuronal and glial cell activation resulting in the release of neurotoxic factors such as inflammatory cytokines or free radicals [28,29]. The chronic administration of LPS can cause spatial memory and learning impairment analogous to cognitive decline during AD, which is associated with inflammation and amyloidogenesis due to increased Aβ deposition [30,31,32]. In the present study, we found that krill oil decreased amyloidogenesis and memory deficiency via the prevention of brain damage by oxidative stress and neuroinflammation. We also found that the krill oil components EPA and DHA reduced LPS-induced oxidative stress and inflammatory response in BV-2 cells. 

Recent studies revealed that Aβ production plays a major role in regulating microglial ROS generation in during AD [33,34]. Hence, oxidative stress leading to attack by free radicals on neural cells increases lipid peroxidation, subsequently causing neurodegenerative conditions such as AD [25]. In our study, krill oil inhibited LPS-induced lipid peroxidation as well as H_2_O_2_ generation, and these inhibitory effects were associated with reduced Aβ accumulation level. The promoters of APP and BACE1 contain NF-κB consensus sequences, which control the transcription of these genes [21]. NF-κB is activated by inflammatory mediators and oxidative stress [35]. Thus, the inhibitory effect of krill oil on NF-κB could be associated with its overall anti-amyloidogenic property owing to its anti-inflammatory and anti-oxidative effects. In our previous study, l-theanine, EGCG, and punicalagin, which are antioxidant compounds, showed anti-neuroinflammatory responses and anti-amyloidogenic activity through antioxidative mechanisms [36,37,38]. The findings of several studies suggest that patients with mild Alzheimer’s deterioration could benefit from taking dietary supplement formulation containing both the omega-3 fatty acids, EPA and DHA [39]. The high content of the two biologically active components EPA and DHA are responsible for the majority of physiological effects of krill oil [40]. EPA or DHA intake resulted in an increased incorporation of omega-3 fatty acids in membrane phospholipids of immune cells; they can be absorbed quickly, cross the BBB, and reduce inflammatory responses as well as the activation of microglia in the brain [41]. EPA and DHA can modulate the expression of several inflammatory genes such as COX-2 and iNOS by significantly reducing NF-κB activity, which subsequently lowers the induction of inflammation and oxidative stress in cells [42,43]. In the present study, LPS-induced phosphorylation of IκB and translocation of p50 and p65 were significantly decreased by treatment with EPA or DHA. Furthermore, EPA and DHA reduced the increased level of LPS-induced oxidative stress and neuroinflammatory gene expression. Thus, antioxidative and anti-inflammatory properties of krill oil could be significant for anti-amyloidogenesis through reducing NF-κB activation, and this effect could be associated with the effects of EPA and DHA. 

Krill consumption by humans can potentially help healthy nutrition strategy to protect against progressive cognitive loss [2]. Taken together, these data indicate that antioxidative, anti-neuroinflammatory, and anti-amyloidogenic effects of krill oil could enhance memory function. Hence, krill oil can be employed for the development of functional food or drug for treating AD. 

## 4. Methods

### 4.1. Ethical Approval

The experimental protocols (27 March 2017) were carried out according to the guidelines for animal experiments of the Institutional Animal Care and Use Committee (IACUC) of Laboratory Animal Research Center at Chungbuk National University, Korea (CBNUA-1073-17-01). All efforts were made to minimize animal suffering and to reduce the number of animals used. All mice were housed in three mice per cage with automatic temperature control (21–25 °C), relative humidity (45–65%), and 12 h light-dark cycle illuminating from 08:00 a.m. to 08:00 p.m. Food and water were available ad libitum. They were fed pellet diet consisting of crude protein 20.5%, crude fat 3.5%, crude fiber 8.0%, crude ash 8.0%, calcium 0.5%, phosphorus 0.5% per 100 g of the diet (obtained from Daehan Biolink, Chungcheongbuk-do, Korea). During this study, all mice were especially observed for the normal body posture, piloerection, ataxia, urination, etc. twice daily to minimize their pain and discomfort.

### 4.2. Materials

#### 4.2.1. Preparation of Enzymatically Decomposed Krill Oil

The Antarctic krill oil was supplied from Alpha B&H (Eumseong-gun, Chungcheongbuk-do, Korea) and we feed the rodent chow supplemented with 5 wt % of krill oil (2018 Teklad Rodent Diet, Envigo Bioproducts) ad libitum. Enzymatically decomposed Krill oil was prepared as reported previously and stored at room temperature until use. Briefly, frozen or freeze-dried krill were thawed. Salt was removed from the krill by washing with tap water, and then they were pulverized using a pin-type mill. Pulverized krill (62.29%, *w*/*w*) were mixed with alcalase enzyme (0.19%, *w*/*w*), a non-specific subtilisin-related serine protease separated from *Bacillus licheniformis*, and water (37.52%, *w*/*w*), then stirred for 30–60 min at room temperature. Before performing the enzyme reaction, the pH of the krill was adjusted to be 7.5–9.0. The enzyme reaction was performed at 57 ± 3 °C for 3.5 ± 0.5 h until liquefied. After performing the enzyme reaction, the pH of the reactant was adjusted to 4.5 ± 0.5 by adding 1.84 part by weight citric acid and/or ascorbic acid per 100 parts by weight liquefied krill and letting stand for 30 min. The enzymes were inactivated by heating at 94 ± 5 °C. The sludge including shell and head of krill was removed by decanter centrifugation (3.0 t/h) at >70 °C. The lipids and phospholipids of filtrate were extracted by centrifugation at 5000 rpm (1.0–2.0 t/h). The extract was sterilized and concentrated under reduced pressure on a rotary evaporator at 80–90 °C until the water content has dropped below 3%. The sterilized concentrate was filtered using 50 mesh sieve and was stored at room temperature until use.

The main components in krill oil are about 7% docosapentaenoic acid (C22:6, DHA) and 12% eicosapentaenoic acid (C20:5, EPA). Furthermore, we purchased EPA and DHA from TOCRIS. The EPA and DHA (final concentration of 100 mM) were dissolved in 100% dimethyl sulfoxide (DMSO), and aliquots were stored at −20 °C until use in vitro. The LPS was purchased from Sigma (serotype O55:B5, Sigma, St. Louis, MO, USA). The LPS (final concentration of 1 mg/mL) was dissolved in PBS, and aliquots in PBS were stored at −20 °C until use.

#### 4.2.2. Animal Experiment

**Figure 7:**
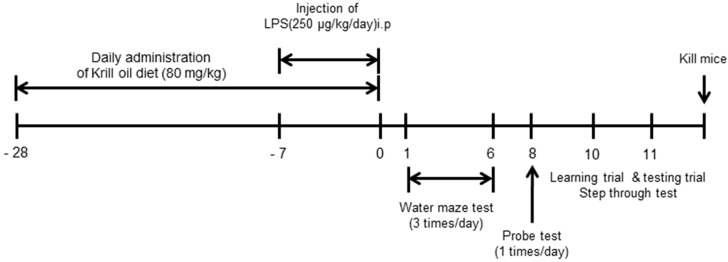
Timeline depicts the treatment of Krill oil and assessments of cognitive functions of mice.

Eight- to ten-week-old male imprinting control region (ICR) mice (Daehan Biolink, Chungcheongbuk-do, Korea) were maintained and handled in accordance with the humane animal care and use guidelines of Korean FDA. ICR mice were randomly divided into three groups: (I) Control group; (II) LPS group; and (III) Krill oil + LPS group. Each group was assigned 10 mice. The Krill oil diet (80 mg/kg) was given to (III) group daily for 4 weeks. Intraperitoneal (i.p.) injection of LPS (250 µg/kg) was administered except for control group on the 4th week for 7 days. Control mice were given an equal volume of vehicle instead. The behavioral tests of learning and memory capacity were assessed using water maze, probe and passive avoidance test. Mice were sacrificed after behavioral tests by CO_2_ asphyxiation (Figure 7).

#### 4.2.3. Behavior Tests

Memory test was performed by the Morris’s water maze test as described elsewhere with SMART-CS (Panlab, Barcelona, Spain) program and equipment [44]. The platform was removed from the pool which was used in the water maze test, and the mice were allowed to swim freely. The swimming pattern of each mouse was monitored and recorded for 60 s using the SMART-LD program (Panlab). Retained spatial memory was estimated by the time spent in the target quadrant area. The passive avoidance response was determined using a “step-through” apparatus (Med Associates, Georgia, VT, USA). All three behavior test were done as described elsewhere [44].

#### 4.2.4. Brain Collection and Preservation

After behavioral tests, mice were perfused with phosphate-buffered saline (PBS) with heparin under inhaled CO_2_ anesthetization. The brains were immediately removed from the skulls and divided into left and right hemisphere. One was stored at −80 °C, while the other was fixed in 4% paraformaldehyde for 72 h at 4 °C and transferred to 30% sucrose solutions. 

#### 4.2.5. Immunohistochemical Staining

Immunohistochemical staining was performed as described previously [45]. The sections were incubated overnight with a rabbit/mouse polyclonal antibody against GFAP; SC-33673 (1:300, Santa Cruz Biotechnology Inc., Santa Cruz, CA, USA), IBA-1; NB100-1028, iNOS; NB300-605 (1:300; Novus Biologicals, Inc., Littleton, CO, USA), COX-2; #12282 (1:300; Cell Signaling Technology, Inc., Beverly, MA, USA). To prevent nonspecific staining, a blocking step was included. Sections were incubated at room temperature for 2 h with 5% bovine serum albumin [46] (in PBS), and incubated for overnight at 4 °C with the primary antibody in blocking solution (5% BSA). Immunohistochemical staining was performed on 8 mice per group (3 sections per each mouse).

#### 4.2.6. Immunofluorescence Staining

The microglial BV-2 cells were incubated for 2 h at room temperature with a goat polyclonal antibody against p65 (1:500, Santa Cruz Biotechnologies, Inc., Santa Cruz, CA, USA). After washing with PBS, the brain sections were incubated with an anti-rabbit or anti-mouse secondary antibody labeled with Alexa-Fluor 488 for 2 h at room temperature. Sections were then dehydrated in ethanol, cleared in xylene and covered with Permount. Final images were acquired using a confocal laser scanning microscope (TCS SP2, Leica Microsystems AG, Wetzlar, Germany).

#### 4.2.7. Western Blot Analysis

We extracted total protein by total lysis buffer (iNtRON Biotechnology, 17081). Furthermore, we used nuclear extraction kit (Abcam, ab113474, Cambridge, MA, USA) for obtaining nuclear protein. In the in vivo study, to compare the expression of protein levels through Western blotting, we selected and used 3 of 10 mice brain from each group. An equal amount of total protein (20 µg) was resolved on 8–15% sodium dodecyl sulfate-polyacrylamide gel and then transferred to a nitrocellulose membrane (Hybond ECL; Amersham Pharmacia Biotech, Piscataway, NJ, USA). To detect target proteins, specific antibodies against APP; NB110-55461, IBA-1; NB100-1028, iNOS; NB300-605 (1:1000, Novus Biologicals, Inc., Littleton, CO, USA), BACE1; #5606, COX-2; #12282 (1:1000, Cell Signaling Technology, Inc., Beverly, MA, USA), GFAP; SC-33673 and β-actin; SC-47778 were used. The blots were then incubated with the corresponding conjugated goat anti-rabbit; SC-2004 or goat anti-mouse; SC-2005 or donkey anti-goat; SC-2020 IgG-horseradish peroxidase (HRP) (1:5000; Santa Cruz Biotechnology Inc., Santa Cruz, CA, USA) secondary antibodies. Immunoreactive proteins were detected with an enhanced chemiluminescence Western blotting detection system. The relative density of the protein bands was scanned by densitometry using MyImage (SLB, Seoul, Korea) and quantified by Labworks 4.0 software (UVP Inc., Upland, CA, USA).

#### 4.2.8. Nitrate Assay

Microglial BV-2 cells were plated at a density of 5 × 10^5^ cells/well in 6-well plates per 2 mL medium for 24 h. After removing the culture medium, the cells were then treated with LPS (1 µg/mL) and EPA, DHA (50, 100 µM) per 2 mL medium for 24 h. The nitrite in the supernatant was assessed using a NO detection kit (iNtRON Biotechnology, Seongnam, Korea), according to the manufacturer’s instructions. Finally, the resulting color was assayed at 520 nm using a microplate absorbance reader (VersaMax ELISA, Molecular Devices, Sunnyvale, CA, USA).

#### 4.2.9. RNA Isolation and Quantitative Real-Time RT-PCR

Tissue RNA was isolated from homogenized hippocampus using RiboEX (Gene All, Seoul, Korea), and total RNA (0.2 µg) was reverse-transcribed into cDNA according to the manufacturer’s instructions using Applied Biosystems (Foster City, CA, USA). For the quantitative, real-time, reverse transcriptase polymerase chain reaction (PCR) assays, the linearity of the amplification of IL-6, IL-1β, TNF-α and β-actin cDNAs was established in preliminary experiments. All signal mRNAs were normalized to β-actin mRNA. cDNAs were amplified by real-time PCR in duplicate with QuantiNova SYBR green PCR kit (Qiagen, Valencia, CA, USA). Each sample was run with the following primer sets: IL-6, 5′-GAGGATACCACTCCCAACAGACC-3′ (sense), 5′-AAGTGCATCATCGTTGTTCATACA-3′ (antisense); IL-1β, 5′-GTGGCTAAGGACCAAGACCA-3′ (sense), 5′-TACCAGTTGGGGAACTCTGC-3′ (antisense); TNF-α, 5′-GATCTCAAAGACAACCAACATGTG-3′ (sense), 5′-CTCCAGCTGGAAGACTCCTCCCAG-3′ (antisense); β-actin: 5′-TGGAATCCTGTGGCATCCATGAAAC-3′ (sense), 5′-TAAAACGCAGCTCAGTAACAGTCCG-3′ (antisense).

#### 4.2.10. Measurement of Aβ_1–42_

Lysates of brain tissue were obtained through protein extraction buffer containing a protease inhibitor. Aβ_1–42_ levels were determined using each specific ELISA Kit (CUSABIO, College Park, MD, USA). Protein was extracted from brain tissues using a protein extraction buffer (PRO-PREPTM, Intron Biotechnology, Korea), incubated on ice for 1 h and centrifuged at 13,000× *g* for 15 min at 4 °C. In brief, 100 μL of sample was added into a pre-coated plate and incubated for 2 h at 37 °C. After removing any unbound substances, a biotin-conjugated antibody specific for Aβ_1–42_ was added to the wells. After washing, avidin conjugated Horseradish Peroxidase (HRP) was added to the wells. Following a wash to remove any unbound avidin-enzyme reagent, a substrate solution was added to the wells and color developed in proportion to the amount of Aβ_1–42_ bound in the initial step. The color development was stopped and the intensity of the color was measured using a microplate absorbance reader (Sunrise™, TECAN, Männedorf, Switzerland).

#### 4.2.11. Oxidative Stress Assay

Hydrogen peroxides were measured according to the manufacturer’s instructions (Cell Biolabs, San Diego, CA, USA). Malondialdehyde (MDA) and hydrogen peroxide were measured according to the manufacturer’s instructions (Cayman Chemical, Ann Arbor, MI, USA). To perform the assay, the brain tissues were homogenized, then normalized to protein concentration. Superoxide production in the brain was detected by dihydroethidium staining (Sigma-Aldrich). Brains were incubated with 5 µM DHE for 30 min at 37 °C in a humidified chamber protected from light. The average fluorescence intensity of the nuclei was then analyzed using Image Pro-Plus software (Media Cybernetics, Inc., Rockville, MD, USA).

#### 4.2.12. Assay of β-Secretase Activities

β-secretase activity in the mice brains was determined using a commercially available β-secretase activity kit (Abcam, Inc., Cambridge, MA, USA) using a fluorescence spectrometer (Gemini EM, Molecular Devices, CA, USA) as described elsewhere [47].

#### 4.2.13. Microglial BV-2 Cell Culture

Microglial BV-2 cells were maintained with serum-supplemented culture media of DMEM supplemented with FBS (10%) and penicillin (100 units/mL). The microglial BV-2 cells were incubated in the culture medium in a humidified incubator at 37 °C and 5% CO_2_. The cultured cells were treated simultaneously with LPS (1 µg/mL) and several concentrations (50, 100 µΜ) of EPA and DHA dissolved in DMSO.

#### 4.2.14. Statistical Analysis

For the measurement of the image data, ImageJ (Wayne Rasband, National Institutes of Health, Bethesda, MD, USA) was used. For the measurement of the image data, ImageJ (Wayne Rasband, National Institutes of Health, Bethesda, MD, USA) was used. Group differences were analyzed by one-way ANOVA followed by Bonferroni’s post-hoc analysis using GraphPad Prism 5 software (Version 5.02, GraphPad Software, Inc., La Jolla, CA, USA).
